# Socio-demographic predictors of obesity among women in Mukono Central Division in Central Uganda: a cross-sectional study

**DOI:** 10.1186/s12905-023-02679-4

**Published:** 2023-11-06

**Authors:** Justine Athieno, Georgina Seera, Faith Muyonga Mayanja Namayengo, Joweria Nambooze Galabuzi, Mariam Namasaba

**Affiliations:** 1 Mbale Regional Referral Hospital, Mbale, Uganda; 2 The Center for African Area Studies, Kyoto University, Kyoto, Japan; 3 Department of Nutritional Science and Dietetics, Kyambogo University, Kampala, Uganda; 4 Department of Psychology, Kyambogo University, Kampala, Uganda

**Keywords:** Obesity, Total body fat percentage (TBF%), Socio-demographic predictors, Working status, Employment status, Unemployment, Informal employment, Urban, Women, Food security

## Abstract

**Background:**

There is a steadily increasing trend in obesity globally and in Sub-Saharan Africa that disproportionately affects women in most places. This is not different in Uganda, where the Uganda Demographic and Health Survey indicated an increase in obesity among women of reproductive age as measured by the body mass index (BMI). However, studies on the predictors of obesity in women are still limited. Particularly, studies using specific indicators of body fat are scant. This study explored the socio-demographic predictors of obesity as indicated by total body fat percentage among women in the age range of 18 to 69 years old living in Mukono Central Division in Central Uganda.

**Methods:**

A cross sectional study design using quantitative methods was employed. A total of 384 women between 18 and 69 years old from Mukono Central Division in Central Uganda were randomly recruited. A structured questionnaire was used to collect socio-demographic data including age, level of education, marital status, childbearing status, household expenditure, household size and employment status. Total body fat percentage, the indicator for obesity was measured using the body composition meter from TANITA. The data was analyzed using multinomial logistic regression analysis using SPSS version 20.

**Results:**

155 women, nearly two fifths (40.4% CI 95% 38.4–42.4) were classified as obese. Age, marital status, childbearing status, and employment status were the factors that were associated with obesity among these women. Employment status was the only variable that remained significantly associated with obesity among the women after adjusting for other factors. Unemployed women were nearly two times more likely to be obese than the employed women (AOR 1.9; 95% CI 1.1–3.1). The prevalence of obesity among the unemployed and employed women was 48.2% and 34.4% respectively.

**Conclusions:**

Obesity in women was predicted by employment status. An in-depth study on factors that predispose unemployed women to obesity, will be instrumental in guiding interventions to curb the emerging obesity epidemic in Uganda. In the same vein, strategies to reduce levels of unemployment among women living in urban Uganda are essential for protecting public health from the dimension of reducing obesity levels.

## Background

Obesity is a complex multifactorial condition defined by excessive fat accumulation in the body that presents a risk to health [1]. In adults, it is often indicated by a body mass index (BMI) value that is greater than or equal to 30.0 kg/m^2^ [2] and a total body fat percentage (TBF%) value that is greater than or equal to 36.0% [3]. Obesity usually results from a general imbalance in energy intake compared to energy expenditure [4]. Globally, the prevalence of obesity is on the rise [5] and it disproportionately affects women [6]. The global obesity prevalence increased from 6.1% in 1986 to 13.1% in 2016. In 2016, the global ratio of the prevalence of obesity among females to males was 1.4 [7]. It is further predicted that one billion people globally, including 1 in 5 women will be living with obesity by 2030. Across the African region, 1 in 5 women (20.4%) are predicted to have a BMI ≥ 30 kg/m^2^ by 2030 and 74 million women are at risk of experiencing complications of obesity by 2030 [8].

Obesity is one of the key risk factors for many non-communicable diseases (NCDs), such as Type 2 diabetes, cardiovascular disease, musculoskeletal disorders, cancers, and respiratory problems, among others [1]. NCDs are the largest contributors to the years of life lost due to illness, disability, and premature mortality with about 12 million adults dying each year including women due to complications of obesity or overweight [9]. Furthermore, studies have indicated that maternal obesity decreases the efficacy of contraceptives and increases the risk of ovulatory disorders [10]. It is further associated with negative outcomes of pregnancy including gestational diabetes, pre-eclampsia, an increased miscarriage rate [10, 11], still birth and congenital anomalies [11] as well as higher risk of obesity among their children in later life [10].

The global health observatory data indicated that in Uganda, the prevalence of obesity among women of reproductive age was 8.6%, and the ratio of obesity among women compared to men was as high as 4.8% [12]. Uganda is also experiencing an increasing trend in obesity particularly in women [13–19], and related NCDs which also disproportionately affect women compared to men [20]. Uganda, like other countries that are classified as low and middle-income countries have for long experienced high levels of under-nutrition [13–19, 21, 22]. The increasing trend in obesity presents a challenge to the health care system, which has been traditionally overstretched by under nutrition and now must deal also with obesity related NCDs [20, 23].

Obesity has multiple etiological factors, constituting the outcome of the interaction of biological, genetic, socio-demographic, developmental, environmental, and behavioural factors as in the socio-ecological model (SEM) [24], and obesity systems map [25]. Socio-demographic factors like age [19, 26–28], level of education [19] marital status [26], number of children [28], wealth [19, 26–29], household size [28] being female [27, 29] and urban residence [19, 27–29] are known to be associated with obesity in Uganda. Studies elsewhere in Africa have reported a positive association between urban residence and obesity among women [30], as well as employment/working status [31–39].

This positive association between age and overweight/obesity might be partly explained by biological factors that naturally happen as people get older. Most people experience hormonal changes and changes in metabolism that make it more difficult to stay slim [25].

Marital status is an important predictor of obesity among women in Africa and according to [40], being married increases the likelihood of being overweight or obese. According to a study in Kenya, married and cohabiting respondents showed significant increased risk for obesity at 1.9 times more likely compared to unmarried respondents [41]. A study by Tanwi and others also showed that, ‘being married’ status even for rural women in Bangladesh was positively associated with overweight and obesity [42]. It is likely that marital status is associated with obesity in as far as it’s commonly associated with pregnancy related weight gain accompanying childbearing.

In developed countries, low wealth index is often linked to increased body weight [43, 44]. Whereas findings in developing countries often indicate that those classified as rich tend to have higher likelihood of overweight and obesity [45]. A study in Uganda also found the highest risk of obesity was among women living in the richest households, who had 13 times the risk of obesity in comparison with women living in poor households [46]. They propose that these findings could have reflected the reality that most women living in affluent households in Uganda tend to lead a sedentary lifestyle, often drive, or use public transport systems most of the time, and eat more fatty and refined foods, in a current shift of dietary patterns to meet western or the so-called civilized lifestyle and social class difference in the Ugandan context [46].

In contrast to high income countries, in low-income countries, education level is usually found to be positively associated with obesity in as far as it is correlated with income [47]. For instance, a study in Ghana reported that women with higher education were more likely to be overweight or obese compared to those with no formal education [48] whereas a study in Sweden reported that women with low levels of education were twice as likely to be obese [49]. In Uganda, a higher prevalence of overweight is reported among women with primary, secondary, and tertiary level educational attainment in comparison with those that have no formal education [19].

The World Bank 2011, projects NCDs—which often co-exist alongside increasing prevalence of obesity in populations; particularly as it pertains to the extent of fat accumulation in the body— to account for 46% of all deaths by 2030 [50]. Nonetheless, there are currently no official guidelines for the prevention and management of overweight and obesity in Uganda. Ascertaining the prevalence, highlighting the specific socio-demographic predictors of obesity, suggesting management and prevention strategies of obesity will help in coming up with interventions that will be effective in addressing the increasing trends in obesity among women in Uganda which in turn, is critical in achieving the Sustainable Development Goals (SDGs), particularly the SDG 3 of ensuring healthy lives and promoting well-being for all people at all ages [51].

It’s however noteworthy that there are limited studies that explore the social demographic predictors of obesity in Uganda. Those that are available have combined overweight and obesity and mainly used body mass index (BMI) as the indicator [13–23, 26–29]. It is against this background that this study focuses on the socio-demographic predictors of obesity among women 18–69 years in Mukono, Central Uganda using total body fat percentage as an indicator.

## Methods

### Study design

**Fig. 1:**
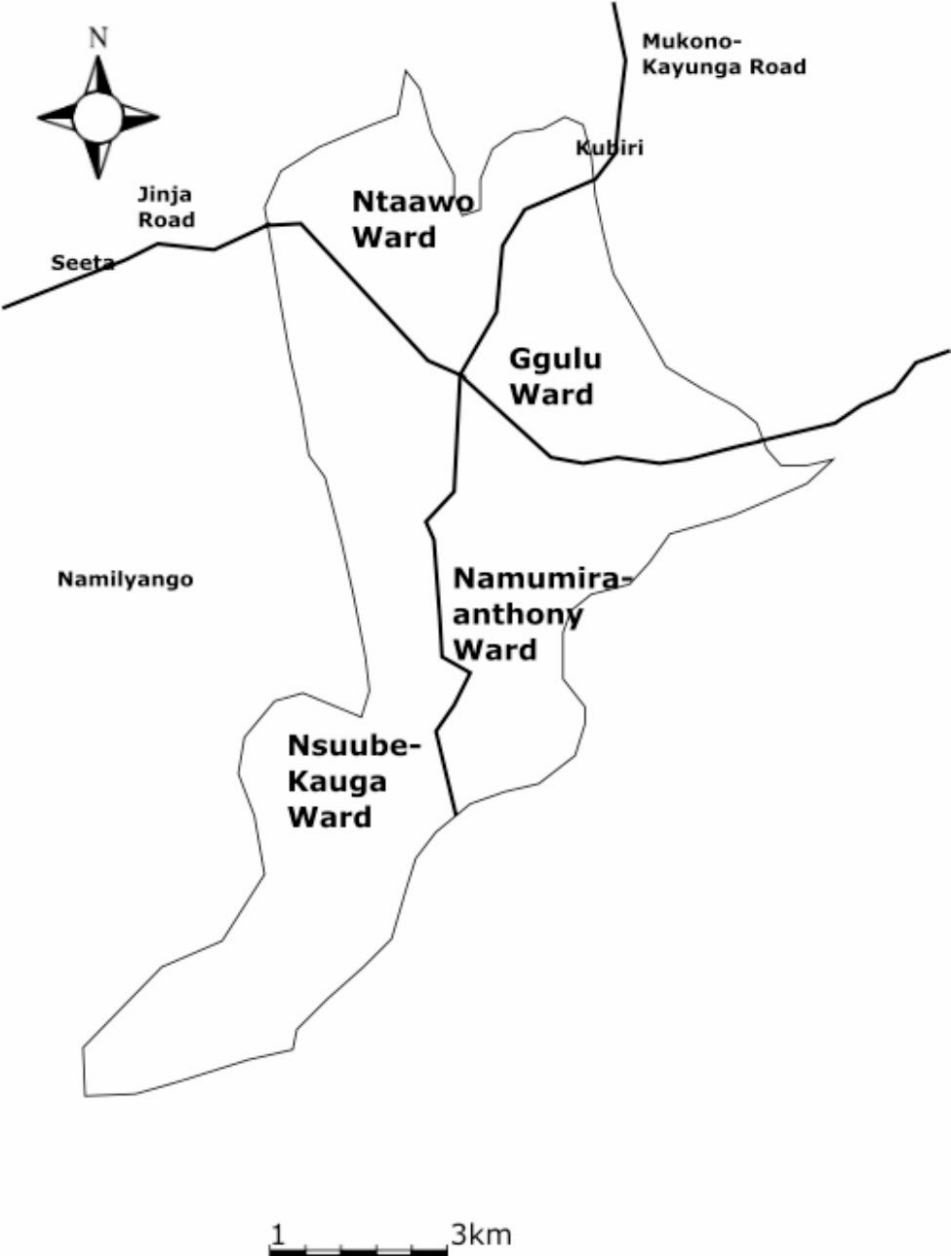
A map of Mukono Central Division showing the four wards/parishes

The aim of the study was to identify the socio-demographic predictors of obesity as indicated by total body fat percentage among women 18–69 years old in Mukono Central Division in Central Uganda. A cross sectional design was used to collect data on the socio-demographic characteristics and obesity status of women. The study was conducted in Mukono Central Division, located in Mukono municipality, which is adjacent to the Capital City, Kampala (Fig. 1).

The division is about 27 kilometres (17 mi) by road in the East of Kampala City. It occupies approximately 31.4 square kilometres (12.1 sq mi) of land area. The coordinates of one of the places in the town of Mukono are 00^o^ 22’ 17.0”N, 32^o^ 44’ 03.7”E [52]. Mukono Central Division was chosen because urban and peri urban areas, particularly those in Central Uganda have been determined in national surveys to have a high prevalence of overweight and obesity in women [19, 29]. Although studies have been carried out in urban Kampala [29] and even in peri urban Eastern Uganda [27], no recent study, to our knowledge has explored the dynamics of obesity in the rapidly urbanizing “greater Kampala” region that includes urban areas in Mukono and Wakiso districts [19].

The study was conducted in August and September of 2018. The study targeted women between 18 and 69 years to facilitate a comparison across a broad range of age groups as did similar studies [27]. Previous studies in Uganda have indicated an increase in the prevalence and incidence of obesity as women grow out of their teens into their twenties and thirties, forties, and upwards [19, 27, 28].

### Inclusion and exclusion criteria

Participants were eligible to participate in this study if they had been residents of the selected villages for at least 6 months and were excluded if they were pregnant or had given birth within 6 months of the study. A minimum residence time was necessary to classify them as residents of the area. Women who had been recently pregnant were excluded because it is difficult to assess body composition with high levels of accuracy during pregnancy and lactation owing to the pregnancy related weight gain that most women experience [53–55].

### Sample size and selection

A total of 384 respondents were included in the regression analysis. The sample size was adequate for the method from the results of the power analysis. A power analysis for a linear multiple regression indicated that the minimum sample size to yield a statistical power of at least 0.8 with an alpha of 0.05 and a medium effect size (d = 0.15) is 103 [56, 57].

The study employed stratified sampling in which a sample of a primary unit was selected and then another sample of secondary units also selected within each primary unit. Mukono Central Division is comprised of 4 wards/parishes. Each of the parishes is comprised of several villages totaling up to 35 [58]. A sampling frame was formed from the 4 parishes/wards: Ntaawo, Ggulu, Namumira-Anthony and Nsuube-Kauga in Mukono Central Division. In the first stage, a raffle was drawn using excel to select one village from each of the parishes and the following villages were selected: Ssaza, Mulago, Kigombya and Nabuti. In the second stage, households in each of the selected villages were visited by the interviewer until the target sample of 384 women was achieved. At each of the households, the interviewer inquired if there was a woman who met the inclusion criteria, informed consent was sought and if she was willing to participate, the woman was interviewed, and her measurements were taken.

### Variables

Socio-demographic factors considered in this study as independent variables included age, marital status, education level, employment status, childbearing status, household expenditure and household size. The dependent variable was obesity status, measured using total body fat percentage (TBF%).

#### Dependent variables

The obesity status of women was measured by total fat percentage (TBF%). Respondent’s age, sex and height were entered into a TANITA BC-202-WH scale. Height was taken with the respondent standing straight with the back of their head, shoulder blades, buttocks, calves, and heels touching the vertical board. The head was positioned to look straight ahead. A thumb and forefinger were placed over the chin to help the respondent to keep the head in an upright position. Using the other hand, the headboard was placed to rest firmly on top of the head and compress the hair. Height was then measured to the last completed 0.1 cm and recorded. To measure TBF, each participant stood (barefooted) on the TANITA scale which uses bio impedance analysis while wearing minimal clothing as recommended by WHO [59]. TBF% values of the participant were displayed by the scale and recorded. The classification of total body fat percentage values of women by age is shown in Table 1.

**Table 1:** Cut offs of total body fat percentage values

Standard cut offs				
Age	Body fat percentage cut off			
	Under fat	Healthy	Over fat	Obese
18 19 20–39 40–59	1–16.9 1–18.9 1–20.9 1–22.9	17–30.9 19–31.9 21–32.9 23–33.9	31–35.9 32–36.9 33–38.9 34–39.9	> or = 36.0 > or = 37.0 > or = 39.0 > or = 40.0
60+	1–23.9	24–35.9	36–41.9	> or = 42.0

Adopted from: TANITA [3]

#### Independent variables

These were the socio-demographic predictors of obesity as shown by various studies [40, 46]. These factors were divided into the individual level (age in years, marital status, employment status and education level in years completed), household level (household size in terms of total number of people) characteristics as was in Sserwanja et al., [26]. Childbearing status in terms of total number of children born to the woman, and household monthly expenditure in Uganda Shillings (UGX) as a proxy for wealth based on the finding from Seera, [28] were also included in the model.

#### Categorization of independent variables

Age was categorized as 0 for 17–19 years old, 1 for 20 to 29 years old, 2 for 30–39 years old, 3 for 40 to 49 years old, 4 for 50 to 59 years old, and 5 for 60 to 69 years old. This is based on the UBOS [19] finding that obesity among women in their thirties and forties is as high as 12.1% and 12.6% respectively while that among women in their twenties and teens is as low as 5.5 and 1.0% respectively.

Marital status was classified as 0 for single/never married, 1 for married/cohabiting, 2 for divorced/separated, and 3 for widowed. This is based on the Kirunda et al., [27] finding that the prevalence of overweight/obesity among people who were married or divorced was as high as 27.5% and 26.7% respectively, while that among those with no marriage experience was 12.6%.

Education was classified as 0 for none, 1 for primary level, 2 for secondary level and 3 for tertiary or university level based on the UBOS report [19]. The prevalence of obesity among those that had only secondary, primary level and no education was 8.7, 5.3 and 6.2% respectively, while the prevalence among those that had tertiary level education was 17.5%.

Childbearing status was classified as 0 for no children and 1 for one or more children. This is based on the findings by Seera [28] that childbearing is associated with overweight and obesity in women.

The monthly expenditure was categorized as 0 for less than 145,052UGX and 1 for more than 145,052UGX based on the UBOS [60] finding that the average daily amount of household expenditure in urban Central Uganda was 145,052UGX.

Similarly, the household size was classified as 0 for less than 4 and 1 for greater than or equal to 4 based on the UBOS [60] finding that the average household size in urban Central Uganda was 4 people.

Lastly, the employment status was classified as 0 for unemployed and 1 for employed/engaged in some kind of income generating activity. This was based on the observation from the current study that a large number of women were unemployed i.e., not engaged in any kind of income generating activity.

### Ethical considerations

The researcher got a recommendation from Mbale Regional Referral Hospital Research and Ethics committee (**No. MRRH-REC OUT01042018).** The research protocol and instruments were submitted to the Uganda National Council for Science and Technology (UNCST) for approval and registration **(No. SS4961).** Clearance was sought from the town clerk of Mukono Central Division. The offices of the LC1 chairpersons of each of the selected villages were visited to obtain permission. Details of the study were explained to the respondents and written informed consent was sought from each respondent who agreed to participate in the study.

### Data analysis

The data were analyzed quantitatively using SPSS Version 20. Frequency distribution analyses, chi square test analysis, and multinomial logistic regression analyses were performed to identify the predictors of obesity as classified by TBF%. Marital status and employment status were entered into the model as categorical variables. The rest of the variables including age, education level, number of children, household expenditure and household size were entered into the model as continuous variables. Multicollinearity was assessed using the Tolerance and Variance inflation factor (VIF) statistics by linear regression analysis. As described by Field [61] absence of multicollinearity was concluded based on four major results (i) the largest VIF was not greater than 10, (ii) the average VIF was not substantially greater than 1, there was no tolerance value below 0.2 or 0.01. A result was considered significant if the p value was less than 0.05. Prevalence and all results are presented for analysis at the 95% confidence interval.

## Results

### Socio-demographic characteristics of women

The study included a total of 384 women. Of these, 90 (23.4%) were residents of Nabuti village in Nsuube-Kauga Parish; 99 (25.8%) were residents of Kigombya village in Namumira-Anthony parish; 119 (31.0%) were residents of Mulago village in Ntaawo parish; and 76 (19.8%) were residents of Ssaza village in Ggulu parish, respectively. Table 2 shows the socio-demographic characteristics of the 384 respondents.

**Table 2:** Socio-demographic characteristics of respondents (n = 384)

Characteristics	Categories	(n)	(%)
Age (years)	17–19	32	8.3
	20–29	195	50.8
	30–39	88	22.9
	40–49	33	8.6
	50–59	32	8.3
	60–69	4	1.0
Education Level	None	22	5.7
	Primary	106	27.6
	Secondary	213	55.5
	Tertiary and University	43	11.2
Marital status	Single- Never married	103	26.8
	Married/Cohabiting	217	56.5
	Divorced/Separated	50	13.0
	Widowed	14	3.6
Childbearing status	No child	73	19.0
	At least one child	311	81.0
Employment status	Unemployed	166	43.2
	Formal employment	5	1.3
	Informal employment	202	52.6
	Casual laborer	2	0.5
	Commercial agriculture	9	2.3
Expenditure category	Below average (UGX145,052)	82	21.4
	Above average (UGX145,052)	302	78.6
Household size	Below average (< 4)	221	57.6
	Above average (≥ 4)	163	42.4

More than half of the respondents (50.8%) were in the 20–29 years old age group. Many of the study participants (55.5%) had secondary level education. Several were currently married or cohabiting (56.5%). Most of the participants had at least one child (81.0%). 56.8% were employed or engaged in some kind of income generating activity. 78.6% were in the expenditure category above the average in urban Uganda. Lastly, 57.6% of the women lived in households with less than four people.

### Prevalence of obesity

The prevalence of obesity based on TBF% was 40.4% (155 women). In addition, 24.0% were classified as overfat (92 women). Only 126 (32.8%) of the women were classified as “healthy”, while 11 (2.9%) were classified as under fat.

### Factors associated with obesity

Age, marital status, parenthood, and employment status were the factors that were associated with obesity among these women (Table 3). The highest prevalence of obesity was observed among women in the 17–19 age group, as well as women in the 50–59, 60–69 and those in the 30–39 age group. The highest prevalence of obesity was observed among women who were currently married/cohabiting, followed by those who were widowed and those who were divorced or separated. The lowest prevalence was observed among those that were single and had never been married. The prevalence of obesity was much higher among those that had at least one child than among those that had no child.

**Table 3:** Factors associated with total bod fat percentage-defined obesity of women

Characteristic (X^2^, df, p)	Category	Under fat (%)	Healthy (%)	Overfat (%)	Obese (%)	Total
Age* (46.6, 15, 0.000)	17–19	0 (0%)	6 (18.7%)	2 (6.2%)	24 (75.0%)	32 (100%)
	20–29	7 (3.6%)	87 (44.6%)	42 (21.5%)	59 (30.2%)	195 (100%)
	30–39	2 (2.3%)	18 (20.4%)	27 (30.7%)	41 (46.6%)	88 (100%)
	40–49	0 (0%)	10 (30.3%)	10 (30.3%)	13 (39.4%)	33 (100%)
	50–59	2 (6.2%)	5 (15.6%)	9 (28.1%)	16 (50.0%)	32 (100%)
	60–69	0 (0.0%)	0 (0.0%)	2 (50.0%)	2 (50.0%)	4 (100%)
Education (3.4, 9, 0.945)	None	0 (0.0%)	6 (27.3%)	6 (27.3%)	10 (45.4%)	22 (100%)
	Primary	4 (3.8%)	35 (33.0%)	25 (23.6%)	42 (39.6%)	106 (100%)
	Secondary	7 (3.3%)	72 (33.8%)	51 (23.9%)	83 (39.0%)	213 (100%)
	Tertiary and University	0 (0.0%)	13 (30.2%)	10 (23.2%)	20 (46.5%)	43 (100%)
Marital status* (20.0, 9, 0.018)	Single	3 (2.9%)	48 (46.6%)	24 (23.3%)	28 (27.2%)	103 (100%)
	Married/cohabiting	5 (2.3%)	63 (29.0%)	49 (22.6%)	100 (46.1%)	217 (100%)
	Divorced/separated	2 (4.0%)	14 (28.0%)	13 (26.0%)	21 (42.0%)	50 (100%)
	Widowed	1 (7.1%)	1 (7.1%)	6 (42.8%)	6 (42.8%)	14 (100%)
Parenthood* (11.5, 3, 0.009)	No child	4 (5.5%)	34 (46.6%)	15 (20.5%)	20 (27.4%)	73 (100%)
	At least one child	7 (2.2%)	92 (29.6%)	77 (24.7%)	135 (43.4%)	311 (100%)
Employment* (24.1, 12, 0.02)	Unemployed	3 (1.8%)	50 (30.1%)	33 (19.9%)	80 (48.2%)	166 (100%)
	Formal	1 (20.0%)	2 (40.0%)	0 (0.0%)	2 (40.0%)	5 (100%)
	Informal	6 (3.0%)	73 (36.1%)	54 (26.7%)	69 (34.1%)	202 (100%)
	Casual labourer	0 (0.0%)	1 (50.0%)	0 (0.0%)	1 (50.0%)	2 (100%)
	Commercial agriculture	1 (11.1%)	0 (0.0%)	5 (55.5%)	3 (33.3%)	9 (100%)
Expenditure (0.2, 3, 0.974)	Below average	2 (2.4%)	26 (31.7%)	21 (25.6%)	33 (40.2%)	82 (100%)
	Above average	9 (3.0%)	100 (33.1%)	71 (23.5%)	122 (40.4%)	302 (100%)
Household size (5.4, 3, 0.144)	Average	8 (3.6%)	75 (33.9%)	44 (19.9%)	94 (42.5%)	221 (100%)
	Above average	3 (1.8%)	51 (31.3%)	48 (29.4%)	61 (37.4%)	163 (100%)

*p < 0.05, **p < 0.01

The prevalence of obesity was highest among those that were “casual labourers” (50.0%) i.e., were basically unemployed and only engaged in income generating activities when they had a chance e.g., those that were invited by neighbors to wash their clothes in exchange for a pay. This was closely followed by the unemployed women, with a prevalence of 48.2%, and those engaged in formal employment with a prevalence of 40%. The prevalence of obesity was lowest among those that engaged in commercial agriculture and those that engaged in informal employment activities; 33.1% and 34.1%, respectively. The highest prevalence of obesity was observed in the group of women that had tertiary or university level education, but also among women that had no education.

The prevalence of obesity was similar among women who had average daily expenditure similar to that observed in urban Uganda, and those that had an average expenditure that was lower than the average observed in urban central Uganda. The prevalence of obesity seemed to be lower in households that had a number of people that is less than the average observed in urban central Uganda.

### Predictors of obesity

Results for the predictors of obesity based on multinomial logistic regression analysis are shown in Table 4. Unemployed women were 1.9 times more likely to be obese than working women (AOR 1.870; 95% CI 1.1160–3.133).

**Table 4:** Predictors of total body fat percentage-defined obesity of women

Independent variable	95% CI for odds ratio (OR)			
*Obese vs. Healthy/Under fat*	Odds Ratio (OR)	Lower	Upper	B(SE)
Intercept				-0.863 (1.208)
Age (Years)	1.033	0.997	1.069	0.032 (0.018)
Education (Years)	1.018	0.950	1.019	0.018 (0.035)
Marital status-Single	0.385	0.063	2.344	-0.953 (0.921)
Marital status-Married/Cohabiting	0.765	0.130	4.508	-0.267 (0.905)
Marital status-Divorced/Separated	0.752	0.119	4.762	-0.285 (0.942)
Children (Number)	1.037	0.871	1.235	0.036 (0.089)
Employment status-Unemployed	1.870	1.116	3.133	0.626 (0.263)*
Expenditure (UGX)	1.000	1.000	1.000	0.000 (0.000)
Household size (Number)	0.938	0.825	1.067	-0.064 (0.065)

*Note*: R^2^ = 0.100 (Cox and Snell), 0.113 (Nagelkerke). Model X^2^ (18) = 39.392, p < 0.003

*p < 0.05, **p < 0.01, OR = Odds ratio at 95% CI OR > 1 = high likely, = 1 = equal, < 1 = less likely

### Prevalence of obesity among women engaged in specific occupation activities

The prevalence of obesity among women engaged in the different occupation activities identified in the study is shown in Table 5. The lowest prevalence of obesity was observed among women working in restaurants or operating food stalls (0.0%); those working as waitresses or bar attendants (16.7%); and those working as “businesswomen” – typically juggling several different activities in efforts to make a living (20.0%). This was followed by those that worked as casual workers – typically washing clothes for others (33.3%); those who worked as hardware shop attendants (33.3%); those who worked as moulders or charcoal stove makers (33.3%); and those who worked as secretaries, typically in a stationary or stationary shop where their work regularly involved long standing hours of photocopying (33.3%).

**Table 5:** Specific occupation activities of women

No.	Occupation	Under fat (%)	Healthy (%)	Overfat (%)	Obese (%)	Total (%)
1	Not employed	3 (1.8%)	50 (30.3%))	33 (20.0%)	80 (48.2%)	166 (100)
2	Shop keeper/canteen/kiosk seller	0 (0.0%)	10 (25.0%)	13 (32.5%)	17 (42.5%)	40 (100)
3	Beautician/salon lady	1 (2.8%)	11 (31.4%)	9 (25.7%)	14 (40.0%)	35 (100)
4	Boutique seller/cloth hawker	0 (0.0%)	4 (26.7%)	5 (33.3%)	6 (40.0%)	15 (100)
5	Tailor	1 (6.7%)	3 (20.0%)	5 (33.5%)	6 (40.0%)	15 (100)
6	Restaurant/ food staller	2 (15.4%)	7 (53.8%)	4 (30.8%)	0 (0.0%)	13 (100)
7	Selling fried snacks	0 (0.0%)	4 (36.4%)	4 (36.4%)	3 (27.3%)	11 (100)
8	Businesswoman	0 (40.0%)	4 (40.0%)	4 (40.0%)	2 (20.0%)	10 (100)
9	Farmer	1 (12.5%)	0 (0.0%)	4 (50.0%)	3 (37.5%)	8 (100)
10	Housemaid	0 (0.0%)	3 (37.5%)	1 (12.5%)	4 (50.0%)	8 (100)
11	Charcoal seller	0 (0.0%)	3 (42.8%)	1 (14.3%)	3 (42.8%)	7 (100)
12	Wash clothes	0 (0.0%)	4 (57.1%)	0 (0.0%)	3 (42.8%)	7 (100)
13	Waitress/bar attendant	1 (16.7%)	2 (33.3%)	2 (33.3%)	1 (16.7%)	6 (100)
14	Market vendor	0 (0.0%)	1 (25.0%)	0 (0.0%)	3 (75.0%)	4 (100)
15	Casual work	0 (0.0%)	2 (66.7%)	0 (0.0%)	1 (33.3%)	3 (100)
16	Hardware shop attendant	0 (0.0%)	1 (33.3%)	1 (33.3%)	1 (33.3%)	3 (100)
17	Moulder/charcoal stove maker	0 (0.0%)	2 (66.7%)	0 (0.0%)	1 (33.3%)	3 (100)
18	Teacher	0 (0.0%)	1 (33.7%)	0 (0.0%)	2 (66.7%)	3 (100)
19	Secretary/Secretarial/Stationary	0 (0.0%)	2 (66.7%)	0 (0.0%)	1 (33.3%)	3 (100)
20	Supermarket attendant	0 (0.0%)	1 (33.3%)	0 (0.0%)	2 (66.7%)	3 (100)
21	Factory worker	1 (33.3%)	2 (66.7%)	0 (0.0%)	0 (0.0%)	3 (100)
22	Cleaner	0 (0.0%)	1 (50.0%)	1 (50.0%)	0 (0.0%)	2 (100)
23	Drug shop seller	0 (0.0%)	1 (50.0%)	0 (0.0%)	1 (50.0%)	2 (100)
24	NGO worker	0 (0.0%)	0 (0.0%)	1 (50.0%)	1 (50.0%)	2 (100)
25	Baker	0 (0.0%)	0 (0.0%)	1 100%)	0 (0.0%)	1 (100)
26	Brick maker	0 (0.0%)	0 (0.0%)	1 (100%)	0 (0.0%)	1 (100)
27	Charcoal burner	0 (0.0%)	0 (0.0%)	0 (0.0%)	1 (100%)	1 (100)
28	Cosmetic seller	0 (0.0%)	1 (100%)	0 (0.0%)	0 (0.0%)	1 (100)
29	Dairy milk attendant	0 (0.0%)	1 (100%)	0 (0.0%)	0 (0.0%)	1 (100)
30	Electrician	0 (0.0%)	0 (0.0%)	0 (0.0%)	1 (100%)	1 (100)
31	Carpenter	0 (0.0%)	0 (0.0%)	1 (100%)	0 (0.0%)	1 (100)
32	Interpreter	0 (0.0%)	1 (100%)	0 (0.0%)	0 (0.0%)	1 (100)
33	Dobi	1 (100%)	0 (0.0%)	0 (0.0%)	0 (0.0%)	1 (100)
34	Trainer (MDD)	0 (0.0%)	0 (0.0%)	0 (0.0%)	1 (100%)	1 (100)
35	Mobile money	0 (0.0%)	1 (100%)	0 (0.0%)	0 (0.0%)	1 (100)
36	Security officer	0 (0.0%)	1 (100%)	0 (0.0%)	0 (0.0%)	1 (100)
37	Technician	0 (0.0%)	1 (100%)	0 (0.0%)	0 (0.0%)	1 (100)
38	Trainer/Coach	0 (0.0%)	1 (100%)	0 (0.0%)	0 (0.0%)	1 (100)
39	Rentals	0 (0.0%)	0 (0.0%)	1 (100%)	0 (0.0%)	1 (100)
	Total	11 (2.9%)	126 (32.8%)	92 (24.0%)	155 (40.0%)	384 (100)

## Discussion

Employment status predicted obesity among the women in this study. The study revealed that unemployed women were more likely to be obese than those who were employed. Evidence on the relationship between employment and obesity status among women living in sub-Saharan countries in Africa and reasons for the observed association has so far been limited and is still largely inconclusive. For instance, Mkuu et al., [31] found similarly to the present study that a non-working status of women was associated with a higher likelihood of being obese in Kenya based on data from a nationally representative sample of the Kenya Demographic and Health Survey (KDHS) [32]. They hypothesized that this observation was associated with the concurrent observation that married women were more likely to be obese, owing to the greater economic security implied by being in a union as opposed to being single.

On the other hand, Pallangyo et al., [33], found that it was current working status that was associated with obesity among women in Tanzania in Dar es Salaam where a convenient sample attending an international trade fair was selected. However, explanation is not offered as to why current working status is associated with obesity in this way in this group of women.

In another study in Tanzania, formally employed subjects were specifically at increased risk of obesity compared to the informally employed subjects. Although, after adjusting for other factors, the difference was not significant [34]. This is likely owing to the very small number of formally employed subjects in their study as compared to the number of informally employed subjects (36 of 362; 10.8%). A pattern which is common in most sub-Saharan African countries, including the present study. They quote Nyaruhucha et al., [35] who reported that occupation was associated with obesity in Tanzania, with formally employed people who were engaged in low energy demanding tasks being more likely to be obese. In addition, the “informal employment” in their study was mostly characterized by agricultural work activities which often involve more exertion and require the utilization of more energy than most formal work.

Another study in the same Capital of Dar es Salaam in Tanzania found that women with informal employment were more likely to be overweight or obese than those who were unemployed. Whereas formal employment had no significant association with obesity [36]. They don’t explain why this may be the case in this group of women.

However, Mukabutera et al., [37] also based on a nationally representative sample of the Rwanda Demographic and Health Survey (RDHS) found that there was no relationship between employment status and obesity [38]. This observation is likely to have occurred because the analysis lumped together participants living in rural and urban areas. This would steer the data towards a direction where most people are engaged in agriculture leaving only a very small population characterized as unemployed, hence the absence of a significant difference. In urban areas however, the livelihood activities of individuals are more diverse, facilitating such differences as those observed in urban Uganda, and Kenya and Tanzania.

In another study in Rwanda that included mostly urban participants, it was observed that unemployed workers and people employed in public sector were more likely to avoid obesity (less likely to be obese) than the workers employed in the informal (private) sector [39]. This is in direct contrast to the findings of the present study, and those of the Kenyan study.

These contrasting findings indicate that the relationship of employment status and obesity could be related to the specific characteristics of the formal or informal job rather than just the fact of it being formal or informal, as well as the specific characteristics of the employment as opposed to no employment as pertains to the known predictors of obesity. This leaves the questions of what the specific characteristics of the daily lives of women who stay at home (unemployed) are, that may predispose them to obesity as in the case of this study. Or what are the characteristics of the daily lives of women engaged in formal or informal employment respectively that may predispose them to obesity as demonstrated by other studies.

The present study contradicts studies in low-income countries in sub-Saharan Africa which usually indicate that employed women are more likely to be overweight/obese—as determined by the body mass index (BMI), due to more income and sedentary lifestyle. In addition, many of the socio-demographic factors that are usually associated with obesity were not significant in this study. A possible reason for this contradiction is that obesity in our study was defined based on levels of total body fat. Furthermore, most employed women in this subgroup were involved in work that increased physical activity and energy expenditure such as tending to small shops—MET value: 2.3 kcal/kg/hour, working in hair dressing salons—2.5 kcal/kg/hour, hawking or selling clothes in a boutique—MET value: 2.5 kcal/kg/hour and tailoring—MET value: 3.5 kcal/kg/hour. In contrast, those who stay at home may have more opportunities for being sedentary, frequently engaging in activities with a MET value of 1.5 kcal/kg/hour or less [63].

Most studies in developing economies and in Africa over the years have continued to highlight BMI-defined obesity as being associated with a high socio-economic status [26, 29, 63]. Since unemployment is more commonly experienced by people of a low socio-economic status, our study is one of the first few that are casting the spotlight on the implied socio-economic inequalities as a predictor of obesity in urban Africa, much like the trend that has been consistently observed in Europe and other areas with predominantly developed economies [64]. Ziraba et al., [65] is another study that pointed out that even in sub–Saharan African countries, the highest increases in the prevalence of BMI-defined obesity over time are observed among the poorest—as high as 50% over a span of 10 years, compared to 7% among the richest subgroups. Simply put, the future obese people, even in sub–Saharan African, will be people with low socio-economic status—particularly those with low education level, income and wealth levels.

A UK-based study by Monsivais et al., [66], found similarly that people who were unemployed tended to become obese particularly women. They also found that this weight gain was apparently independent of diet and physical activity, and thus postulated that weight gains associated with unemployment are triggered by psychosocial mechanisms. Economic insecurity and the psychological response of women to it has been observed to trigger perceptions and practices that facilitate weight gain in a group of Ugandan women in the same setting as that of the present study by [67]. The study highlighted a positive perception of an overweight body size, and a reluctance among women to intentionally control their body size through diet and exercise. This was associated with their present and past experience of fluctuations in food availability and accessibility. Because the women had unstable access to food, they preferred to eat as much as possible whenever possible, a practice which inevitably predisposed them to obesity due to periodic high energy intake and subsequent fat accumulation. An earlier study by the same author, Seera [28], found that although both overweight women and obese women had a higher socio-economic status than the women classified as being normal weight using BMI, the overweight women (who were by implication less fat than the obese women), were of a higher socio-economic status than the obese women. This means that when women are poor, they are more likely to be of normal weight or even underweight. However, as income levels increase, lower income women are more likely to be on the latter end of the spectrum – obese.

It was also found in some places that obesity was predicted by being unemployed, but also underweight was associated with being unemployed [68]. This highlights that the mechanism through which unemployment triggers obesity may be linked with the implications for the nutrition of the individuals involved—causing a non-normal nutrition status that is underweight or obese. A link between employment status and nutrition status is further demonstrated by this study. They propose that there exists a relationship between unemployment and other factors known to predispose people to obesity such as diet and physical activity among others but highlight that these relationships are not necessarily linear and are in fact likely heterogeneous.

In conclusion, the present study showed that TBF%-defined obesity in women was predicted by unemployment. This relationship is likely mediated by the implications for economic security. Nevertheless, previous research elsewhere has highlighted that this relationship is more often heterogenous than linear. As such, identifying what it is about being unemployed, that predisposes women to obesity within the urban Ugandan context, will be instrumental in guiding interventions to curb the emerging obesity epidemic among women in urban Uganda.

The main strength of this study is that it classifies obesity based on a measure of total body fat percentage of women. It is one of very few studies since most studies still rely on the anthropometric indicators of BMI, waist circumference and waist hip ratio for classifying obesity. These indicators, particularly BMI, have recently come under scrutiny for their tendency to underestimate or even overestimate the true prevalence of obesity in groups and individuals.

The main limitation of the study is that it was a cross sectional study. Total body fat percentage of women and their socio-demographic characteristics data was collected at a single point in time. It is possible that the results might have been different if the study had adopted a longitudinal design. In addition, two stage sampling might have introduced sampling error since there was no adjustment in sample for this design effect. This may have affected the estimates presented in the results. Lastly, the Tanita BC-202 body composition meter and the recommended body fat percentage cut offs have not been validated in the setting of the study. It is also possible that the results might have been different if the study had included women who commute for work to the distant places such as in Kampala and Wakiso districts due to long commuting times.

## Data Availability

The datasets used and/or analyzed during the current study are available from the corresponding author on reasonable request.

## Abbreviations

AOR: Associated Odds Ratio
BMI: Body Mass Index
CI: Confidence Interval
DF: Degrees of Freedom
KDHS: Kenya Demographic and Health Survey
MI: Miles
MOH: Ministry of Health (in Uganda)
MRRH: REC–Mbale Regional Referral Hospital Research Ethics Committee
NCDs: Non–Communicable Diseases
RDHS: Rwanda Demographic and Health Survey
SEM: Socio–Ecological Model
SE: Standard Error
SDGs: Sustainable Development Goals
OR: Odds Ratio
TBF: Total Body Fat
UBOS: Uganda Bureau of Statistics
UDHS: Uganda Demographic and Health Survey
UGX: Uganda Shillings
UNCST: Uganda National Council for Science and Technology
VIF: Variance Inflation Factor
WHO: World Health Organization
